# Life Stress Impairs Self-Control in Early Adolescence

**DOI:** 10.3389/fpsyg.2012.00608

**Published:** 2013-01-11

**Authors:** Angela L. Duckworth, Betty Kim, Eli Tsukayama

**Affiliations:** ^1^Department of Psychology, University of PennsylvaniaPhiladelphia, PA, USA

**Keywords:** life events, stress, self-control, impulsivity, adolescence

## Abstract

The importance of self-control to a wide range of developmental outcomes prompted the current investigation of negative life events and self-control in early adolescence. In three prospective, longitudinal studies, negative life events reported by the mother (in Study 1) or child (in Studies 2 and 3) predicted rank-order decreases in self-control over time. In all studies, self-control was measured at two different time points using questionnaires completed by three separate raters, including a classroom teacher who knew the child well and two other raters (parents, caregivers, and/or the child himself/herself). Psychological distress measured in Studies 2 and 3 mediated the deleterious effects of negative life events on self-control. These findings extend prior experimental laboratory research documenting the acute effects of stress on self-control.

## Introduction

It is now well-established that negative life events (e.g., parents divorcing or losing their jobs, close friends moving away) predict increases in symptoms of child and adolescent psychopathology (Grant et al., 2004). Likewise, a substantial literature has confirmed that psychological processes intervene between life events and their deleterious consequences. In particular, subjective appraisals of life events as threatening or overly challenging mediate the deleterious effect of these events on outcomes (Cohen et al., 1988; Monroe, 2008).

In the current investigation, we examine how negative life events influence self-control, defined as the tendency to regulate impulses and resist immediately rewarding temptations in the service of long-term goals.^1^ Rank-order differences in self-control among children of the same age predict a range of important life outcomes, including academic achievement, physical health, risky and criminal behavior, and income, even when controlling for the potential confounds of socioeconomic status and general intelligence (Mischel et al., 1989; Tsukayama et al., 2010; Moffitt et al., 2011; Duckworth et al., 2012). Like other dimensions of temperament and personality, self-control is highly rank-order stable – but far from perfectly so – throughout development (Roberts and DelVecchio, 2000; Caspi et al., 2005). In other words, more self-controlled children by definition surpass their peers at regulating behavioral, attentional, and emotional impulses, but they do not always maintain this advantage over time. Why? One possibility, unexamined by prior research, is that negative life events represent an important category of environmental influence on self-control during development.

For several reasons, early adolescence is a particularly interesting stage of development in which to investigate the effects of life stress on self-control. First, adolescence is a period of heightened brain plasticity, particularly for prefrontal areas (Spear, 2010) thought to underlie control processes (Metcalfe and Mischel, 1999). It stands to reason that this plasticity may potentiate the influence of environmental factors that durably shape these brain structures during this period (see also Thompson-Schill et al., 2009).

Second, early adolescence marks the beginning of a normative increase in the strength of sensation seeking impulses in both boys and girls, leading to higher rates of risky behaviors (e.g., smoking and drug abuse, unplanned pregnancy, dropping out of school) with durable influence on later life outcomes (Steinberg, 2004; Moffitt et al., 2011). Thus, even if the effects of life stress on self-control are transient, they may nevertheless have long-term consequences insofar as temporary impairments in self-control lead to irreversible, life-changing decisions.

Finally, it is during early adolescence that children transition from elementary to middle school, a change which for many children precipitates a downward spiral in academic motivation and effort (Eccles et al., 1991). During this transition, systematic changes in the classroom environment (e.g., greater emphasis on teacher control and fewer opportunities for student decision making) are at odds with developmentally normative psychological changes (e.g., self-perceptions as autonomous, independent decision makers; Damon and Hart, 1982; Eccles et al., 1991). The misfit between educational environment and psychological needs makes early adolescence a vulnerable period during which even temporary decreases in self-control might have a particularly detrimental impact on academic performance.

### Prior research on stress and self-control

Prior studies provide indirect support for the prediction that negative life events impair self-control in early adolescence. In particular, negative life events have been associated with emotion regulation in both children (Swearingen and Cohen, 1985; Schwartz and Proctor, 2000) and young adults (McCarthy et al., 2006). Likewise, conditions of poverty (i.e., inadequate housing, economic insufficiency, and frequent departures of adults from the home) have been associated with higher resting levels of salivary cortisol during the first 4 years of life, and this stress response biomarker, in turn, has been associated with worse performance on tasks of executive function (Blair et al., 2011; Blair and Raver, 2012). In the current investigation, we define self-control as a superordinate construct, encompassing the regulation of all impulses that conflict with an individual’s more valued goals and standards (Magen and Gross, 2010). We therefore operationalize self-control using questionnaires assessing control over attentional impulses (e.g., paying attention to teacher’s instructions), behavioral impulses (e.g., breaking bad habits), and emotional impulses (e.g., controlling temper when arguing with peers). Our prediction, untested in prior research, is that negative life events compromise all modalities of self-control because perceptions of threat and uncertainty should potentiate all types of reactive, immediately gratifying impulses.

Experiments with animals suggest that uncontrollable stress rapidly impairs performance on tasks requiring top-down, prefrontal cognitive control, and concomitant architectural changes in the same brain circuits (Radley et al., 2005; Cerqueira et al., 2007; Arnsten, 2009). Likewise, experimental studies have also shown that uncontrollable stressors impair self-control in human subjects (e.g., Glass et al., 1969; Gardner, 1978; Evans, 1979). However, because experimental research has primarily examined the impact of acute stressors (e.g., unpredictable sound blasts, inescapable electric shocks) on short-term changes in task performance in the laboratory, their generalization to life stress as it is naturalistically experienced on behavior in the real world cannot be assumed.

### The current investigation

We undertook three prospective, longitudinal studies of young adolescents which collectively test the following two main hypotheses: (1) Negative life events predict rank-order decreases in self-control, even when controlling for likely confounds (e.g., socioeconomic status), and (2) the subjective experience of psychological distress mediates the relationship between objective negative life events and rank-order decreases in self-control. In Study 1, we tested the first hypothesis using longitudinal data from a large, national sample of children. In Study 2, we collected data from a socioeconomically and ethnically diverse sample of middle school students to replicate the findings in Study 1 and, further, to test psychological distress as a mediator of the negative life events-self-control relationship. In Study 3, we replicated the mediation model of Study 2 in a separate sample of middle school students, staggering assessments over the academic year so that negative life event checklists were completed prior to perceived stress questionnaires.

Given the need for more methodologically rigorous studies on life stress (Compas et al., 2001), design features of the current investigation aimed at increasing internal and external validity are worth noting at the outset. First, in all three studies, we used three different raters (i.e., teachers paired with parents and/or children themselves) to assess self-control. We did so primarily because multi-source measurement approach increases reliability and validity, with multiple sources contributing complementary information about the behavior or trait of interest (Roberts et al., 2006). A further advantage of this measurement strategy is that the observed associations between self-control and prior life events (reported by only one of the sources who rate the child’s self-control) are less likely to be the artifact of common method variance. Second, as recommended by Grant et al. (2004), we used previously validated inventories to measure life events, completed in Study 1 by mothers and in Studies 2 and 3 by children themselves. Third, to eliminate likely third-variable confounds, we controlled for demographic variables and socioeconomic status in all three studies. Likewise, we controlled for baseline levels of self-control in all three studies in order to estimate the variance explained by life events in *changes* in self-control over the period when life events were experienced. In Studies 1 and 2, we were also able to control for baseline levels of negative events, which effectively controls for potential confounds that covary with chronic adverse circumstances in children’s lives. Finally, we sought to maximize external validity by replicating findings using three different measures of self-control in three separate large, socioeconomically and ethnically diverse samples of children.

## Study 1

Using data from the National Institute of Child Health and Human Development Study of Early Child Care and Youth Development (NICHD-SECCYD), we tested the longitudinal effects of life events on changes in self-control. Specifically, we examined the effect of negative life events experienced during the previous year on self-control, controlling for prior levels of self-control and life events, as well as demographic variables.

### Method

#### Participants and procedure

Participants were children from the NICHD-SECCYD, a longitudinal multi-site study originally designed to examine the effects of various child care arrangements on development. Details of study recruitment and data collection protocols are described on the study’s Web site (https://secc.rti.org/). We included in our final sample 80% of the 1,364 participants in the NICHD-SECCYD for whom relevant data on self-control (*n* = 1,060 and 1,041 in grades 4 and 6, respectively) or life events (*n* = 1,028 and 1,012 in grades 3 and 5, respectively) were collected. Participants in our final sample (*N* = 1,094) were not different from those excluded in terms of gender, ethnicity, and age, *p*s > 0.05. Life events were measured in third and fifth grade, and self-control was measured in fourth and sixth grade. When participants were in third grade, their mean age was 8.63 years (SD = 0.23). Approximately 77% of participants were White, 12% were Black, 6% were Hispanic, and 5% were other ethnicities; 50% were female.

#### Measures

##### Negative life events

Mothers completed the Life Experiences Survey when their children were in the third and fifth grades (LES, Sarason et al., 1978). This questionnaire asked mothers to indicate whether any of 60 life experiences that “sometimes bring about changes in people’s lives” occurred *during the past year*. For each identified event, respondents indicated on a seven-point scale, where −3 = *very negative* and +3 = *very positive*, the impact the event has had on their lives. The number of negative life events (typically, events such as “fired or laid off from job,” “major change in emotional closeness of family,” “divorce”) was calculated as the number of experienced events rated as negative (i.e., lower than zero) by the mother. Distributions were right-skewed. Therefore, prior to analyses, we normalized each distribution by grouping scores into five categories, where 1 = *no life events*, 2 = *1–2 life events*, 3 = *3–5 life events*, 4 = *6–8 life events*, and 5 = *9 or more life events*.

##### Self-control

When participating children were in the fourth and sixth grades, their mothers, fathers (or another caregiver if the father was unavailable), and teachers each completed the Social Skills Rating System (Gresham and Elliot, 1990), which asked raters to report how often the child engaged in specific behaviors on a three-point scale ranging from 0 = *never* to 2 = *very often*. Our own factor analyses as well as independent research on separate samples (Whiteside et al., 2007) failed to replicate the original published factor structure of the SSRS. Therefore, to create mother, father, and teacher ratings of the child’s self-control, we averaged nine items from the parent version of the SSRS for parent ratings and 10 items from the teacher version of the SSRS for teacher ratings; these items were chosen based on theoretical alignment with the construct of self-control and observed relations in separate studies with theoretically predicted outcomes (see Tsukayama et al., 2010; Duckworth et al., 2012). The internal reliabilities for parent and teacher scales at each time point were acceptable and ranged from α = 0.76 to 0.88.

In fourth grade, intercorrelations among mother, father, and teacher ratings ranged from *r*s = 0.34 to 0.52, *p*s < 0.001. In sixth grade, intercorrelations among ratings ranged from *r*s = 0.33 to 0.53, *p*s < 0.001. These associations compare favorably to the meta-analytically derived average correlation of *r* = 0.22 between child self-report and informant ratings and *r* = 0.28 between two different types of informant (e.g., parent/teacher) by Achenbach et al. (1987). We created separate composite self-control scores by averaging standardized (i.e., z-scored) mother, father, and teacher measures at each time point. Following Nunnally (1978), we found the internal reliability of the composite self-control score for both grades to be 0.90. On average across the two time points, approximately 55% of participants had mother, father, and teacher ratings, 34% had two of these scores, 7% had one of the scores, and 4% were missing all three. We averaged the two non-missing scores for participants who were missing one score, and we used the single non-missing score for participants who were missing two scores.

##### Socioeconomic and demographic variables

Data on gender, ethnicity, and birthdate were recorded. We assessed socioeconomic status using income-to-needs ratio (assessed in terms of income compared with the U.S. Census Bureau–defined poverty line) at third grade, which we log-transformed to normalize the distribution.

### Results and discussion

Summary statistics and bivariate correlations are provided in Table 1. On average, mothers reported between 3 and 4 negative life events per year. Negative life events were moderately stable over 2 years, *r* = 0.42, *p* < 0.001. As expected, composite self-control scores during the fourth and sixth grade were highly correlated, *r* = 0.70, *p* < 0.001.

**Table 1:** **Summary statistics and bivariate correlations in Study 1**.

Variable	*M*	*SD*	1	2	3	4	5	6	7	8	9	10
1. Negative life events T_1_ (second–third Grade)^a^	3.37	3.44	–									
2. Negative life events T_3_ (fourth–fifth Grade)^a^	3.07	3.18	0.42***	–								
3. Self-control T_2_ (fourth Grade)	−0.03	0.83	−0.10**	−0.03	–							
4. Self-control T_4_ (sixth Grade)	−0.01	0.80	−0.09**	−0.07*	0.70***	–						
5. Age in third Grade	8.63	0.23	0.00	0.04	0.09**	0.08*	–					
6. Income-to-needs ratio^a^	4.39	3.78	−0.08*	0.01	0.36***	0.34***	0.13***	–				
7. Female	50%		−0.04	0.00	0.17***	0.21***	−0.01	0.00	–			
8. White	77%		0.07*	0.10**	0.21***	0.18***	0.09**	0.30***	0.02	–		
9. Black	12%		−0.08*	−0.11***	−0.24***	−0.19***	−0.06*	−0.33***	0.00	−0.67***	–	
10. Hispanic	6%		0.03	0.03	−0.02	−0.03	−0.01	−0.09**	−0.02	−0.47***	−0.09**	–
11. Other	5%		−0.05	−0.05	−0.02	−0.02	−0.07*	0.00	−0.01	−0.43***	−0.09**	−0.06*

^a^For negative life events and as well as socioeconomic status, the means and standard deviations of raw scores are displayed. For all analyses, including bivariate correlations summarized in this table, we used transformations of these variables (see Method section for detail) whose distributions were approximately normal. *p < 0.05. **p < 0.01. ***p < 0.001.

We fit a path model to test whether the number of negative life events experienced during fifth grade predicted decreases in self-control in sixth grade, controlling for self-control in fourth grade, negative life events experienced in third grade, gender, ethnicity, age, and socioeconomic status. About 19% of children were missing data on one or more variables. We therefore used full information maximum likelihood (FIML) estimation, which is less biased and more efficient than traditional missing data techniques (Enders and Bandalos, 2001; Peters and Enders, 2002).

**Figure 1:**
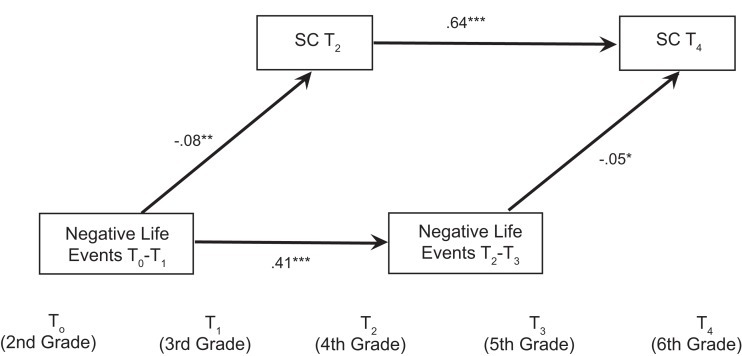
**Standardized path coefficients for path model demonstrating that changes in negative life events predict decreases in self-control in Study 1**. Gender, ethnicity, age, and log-transformed income-to-needs were included as covariates in the model but are not shown. Non-significant paths are also not shown. Life events that occurred during the 1-year period prior to the assessment were reported. SC, Self-control. *p < 0.05, **p < 0.01, ***p < 0.001.

As predicted, changes in negative life events predicted changes in self-control, β = −0.05, *p* = 0.044 (see Figure 1)^2^. Thus, while self-control demonstrated considerable rank-order stability over time, negative life events nevertheless predicted rank-order decreases in self-control.

## Study 2

In Study 1, we found that children who had recently experienced more negative life events were rated lower in self-control by their parents and teachers, controlling for prior ratings of self-control by the same informants. Study 1 had two related limitations, however, both of which stemmed from how negative life events were measured. Using available data in the NICHD-SECCYD dataset, we assessed negative life events as the number of life experiences rated by mothers as negative in valence. When an individual is asked to decide which events qualify as stressful, the distinction between objective life events and perceived stress is blurred (Monroe, 2008). Further, ratings of the valence of events in the NICHD-SECCYD were provided by mothers rather than children. It is possible that a recent life event (e.g., birth of a baby) judged positively by a mother would be judged negatively by her child. Not only were we unable in Study 1 to cleanly separate life events from the psychological distress they caused, we were also constrained by the available data to rely upon mothers’ (as opposed to children’s) reactions to life events. As a consequence, we were unable to test our prediction that psychological distress (i.e., increases in negative affect) mediates the effects of negative life events on self-control.

In Studies 2 and 3, therefore, we measured objective life stressors using the Life Events Checklist (LEC; Johnson and McCutcheon, 1980), an inventory of life events developed and validated for use with older children and adolescents. Like other LECs, the LEC was not designed to be exhaustive but, rather, intended to sample representative significant life events common in childhood and adolescence (Grant et al., 2004). Since Study 1 confirmed our hypothesis that negative life events significantly impacted self-control, we only included life events from the LEC that were unambiguously negative, eliminating items which could be either positive or negative (e.g., moving to a new home). In addition, we eliminated items that confounded impulsive behavior with environmental events (e.g., “increases in number of arguments with parents”) because, as Grant et al. (2004) has pointed out, checklists that include events that are independent of children’s behavior represent “cleaner” markers of environmental effects.

In Study 2, we used a negative affect questionnaire to assess psychological distress. Measures of negative affect and more specifically designed measures of perceived stress have in prior studies been highly correlated (e.g., uncorrected *r* = 0.65, *r* corrected for lack of reliability = 0.77, *p* < 0.001 in Cohen et al., 1993). Moreover, items from standard perceived stress scales (e.g., “In the last month, how often have you felt “nervous and stressed”) are highly similar to items we used to measure negative affect (e.g., “How often in the last month have you felt nervous?”). In sum, in Study 2, we aimed to replicate the association between increased frequency of negative life events and subsequent rank-order decreases in self-control observed in Study 1 and, further, to test psychological distress as a mediator of this relationship.

### Participants

Participants were fifth through seventh grade students at two public schools and one private school in the Northeast. About 92% of the 661 students elected to participate. Of the 610 consented students, 83 were omitted from the analysis because they transferred to other schools or were absent during questionnaire administration. Participants in the final sample (*N* = 527) were not significantly different from excluded participants in terms of gender, ethnicity, or age, *p*s > 0.05. At the first data collection in fall 2008, the mean age of the participants was 11.49 years (*SD* = 1.10). Forty-seven percent of participants were Hispanic, 27% were Black, 21% were White, and 5% were other ethnicities; 53% were female.

### Procedure and measures

Children, parents, and teachers completed consent forms and questionnaires in fall 2008. One year later, in fall 2009, we repeated the same procedure but did not ask parents to fill out questionnaires.

#### Negative life events

We selected 11 age-appropriate items describing negative life events (e.g., “increased arguments or fights between parents,” “close friends had problems”) from the LEC (Johnson and McCutcheon, 1980) and supplemented these with three additional age-appropriate items (e.g., “friends moved away or you moved away from friends”). In fall 2008 and fall 2009, children were asked to indicate whether each of these 14 negative life events occurred in their lives *during the past year*. Acceptable levels of test-retest reliability, convergent validity, and discriminant validity have been reported for the LEC in other samples (Brand and Johnson, 1982).

#### Self-control

In fall 2008, children completed the Impulsivity Scale for Children (ISC; Tsukayama et al., submitted). This questionnaire includes eight items about specific behaviors nominated by children as indicating failures of self-control (e.g., “I did not remember what someone told me to do,” “I interrupted other people while they were talking.”). Children endorsed each item on a frequency scale, where 1 = *almost never*, 2 = *about once a month*, 3 = *about 2–3 times a month*, 4 = *about once a week*, and 5 = *at least once a day*. Separately, one parent and one teacher completed an informant version of the ISC. For consistency with Studies 1 and 3, we reverse-scored each item so that higher scores denote higher self-control.

In a validation study (Tsukayama et al., submitted), the ISC demonstrated convergent validity with the SSRS self-control measure used in Study 1 (*r* = 0.62, *p* < 0.001) as well as the Brief Self-Control Scale (BSCS; Tangney et al., 2004) used in Study 3, *r* = 0.71, *p* < 0.001. The correlation between the SSRS and BSCS measures was *r* = 0.64, *p* < 0.001.

In fall 2009, children completed the ISC a second time. Two of their teachers completed a version of the ISC in which each item of the ISC was rewritten as its obverse, thus denoting acts of self-control (e.g., “This student listened to other students speak without interrupting them”). Teachers rated each child on a frequency scale, where 0 = *0 days out of 5*, 1 = *1 day out of 5*, 2 = *2 days out of 5*, 3 = *3 days out of 5*, 4 = *4 days out of 5*, and 5 = *5 days out of 5*.

In fall 2008, intercorrelations among self-report, parent, and teacher ratings of self-control ranged from *r*s = 0.26 to 0.33, *p*s < 0.001. In fall 2009, intercorrelations among self-report and both teacher ratings of self-control ranged from *r*s = 0.31 to 0.50, *p*s < 0.001. Observed internal reliability coefficients ranged from α = 0.78 to 0.94. We created separate composite self-control scores for fall 2008 and fall 2009 by averaging the mean of standardized scores for the three single report measures. Following Nunnally (1978), we found the internal reliability of composite self-control measures to be 0.92 and 0.95 for fall 2008 and 2009, respectively. On average across the two time points, approximately 68% of participants had all three ratings, 18% had two, and 14% had one. We averaged the two non-missing scores for participants who were missing one score, and we used the single non-missing score for participants who were missing two scores.

#### Negative affect

In fall 2008 and fall 2009, children completed the Positive and Negative Affect Scale for Children (PANAS-C, Laurent et al., 1999), endorsing 15 negative emotions (i.e., sad, frightened, ashamed, upset, nervous, guilty, scared, miserable, jittery, afraid, lonely, mad, disgusted, blue, gloomy) in response to the prompt, “Indicate to what extent have you felt this way during the past month.” The five-point Likert response scale ranges from 1 = *very slightly or not at all* to 5 = *extremely*. Observed internal reliability coefficients for the negative affect subscale in fall 2008 and fall 2009 were α = 0.88 and 0.87, respectively.

#### Socioeconomic status and demographic variables

We obtained data on gender, ethnicity, birthdate, and home addresses from school records. Using home addresses in conjunction with U.S. Census bureau data, we estimated the median household income by census block for each participant and used this estimate as a measure of socioeconomic status.

### Results and discussion

Summary statistics and bivariate correlations are provided in Table 2. Consistent with Study 1, children reported an average of 3–4 negative life events per year. Likewise, the number of annual negative life events was moderately stable over 2 years (*r* = 0.53, *p* < 0.001), and the 1-year test-retest stability of self-control was *r* = 0.59, *p* < 0.001.

**Table 2:** **Summary statistics and bivariate correlations in Study 2**.

Variable	*M*	*SD*	1	2	3	4	5	6	7	8	9	10	11	12	13	14	15
1. Negative life events T_0_–T_1_ (fall 2007–fall 2008)	3.73	2.76	–														
2. Negative life events T_1_–T_2_ (fall 2008–fall 2009)	3.77	2.58	0.53***	–													
3. Negative affect T_1_ (fall 2008)	2.16	0.76	0.37***	0.25***	–												
4. Negative affect T_2_ (fall 2009)	2.04	0.71	0.29***	0.35***	0.44***	–											
5. Self-control T_1_ (fall 2008)	−0.03	0.77	−0.25***	−0.17***	−0.14**	−0.13**	–										
6. Self-control T_2_ (fall 2009)	−0.01	0.84	−0.19**	−0.19***	−0.11*	−0.26***	0.59***	–									
7. Age in fall 2008	11.49	1.10	−0.02	−0.06	0.01	0.02	−0.29***	−0.19***	–								
8. Median household income^a^	$43,626	$37,696	−0.34***	−0.27***	−0.20***	−0.22***	0.05	0.06	0.20***	−							
9. School 1	38%		0.21***	0.18***	0.10*	0.11*	−0.08	−0.01	−0.12**	−0.40***	–						
10. School 2	32%		0.17***	0.15**	0.13**	0.10*	0.05	−0.04	0.12**	−0.36***	−0.54***	–					
11. School 3	30%		−0.39***	−0.34***	−0.24***	−0.21***	0.04	0.05	0.25***	0.79***	−0.51***	−0.45***	–				
12. Female	53%		0.11*	0.05	0.11*	0.09*	0.14**	0.16***	0.02	−0.01	0.01	0.02	−0.02	–			
13. White	21%		−0.37***	−0.34***	−0.22***	−0.23***	0.07	0.07	0.21***	0.71***	−0.41***	−0.36***	0.83***	−0.10*	–		
14. Black	27%		0.24***	0.07	0.03	0.03	−0.20***	−0.14**	−0.07	−0.28***	0.01	0.26***	−0.28***	0.03	−0.32***	–	
15. Hispanic	47%		0.13**	0.23***	0.15**	0.18***	0.14**	0.09*	−0.15**	−0.44***	0.41***	0.12**	−0.57***	0.03	−0.48***	−0.58***	–
16. Other	5%		−0.10*	−0.05	0.01	−0.03	−0.03	−0.05	0.09*	0.23***	−0.18***	−0.12**	0.33***	0.05	−0.12**	−0.14**	−0.21***

*p < 0.05. **p < 0.01. ***p < 0.001.

We fit a path model to test whether the number of negative life events experienced during the prior year predicted rank-order decreases in self-control, controlling for the number of negative life events experienced the year before, school site, gender, ethnicity, age, and socioeconomic status. About 7% of children were missing data on one or more variables; consistent with Study 1, we accounted for missing data by using FIML to estimate path models.

**Figure 2:**
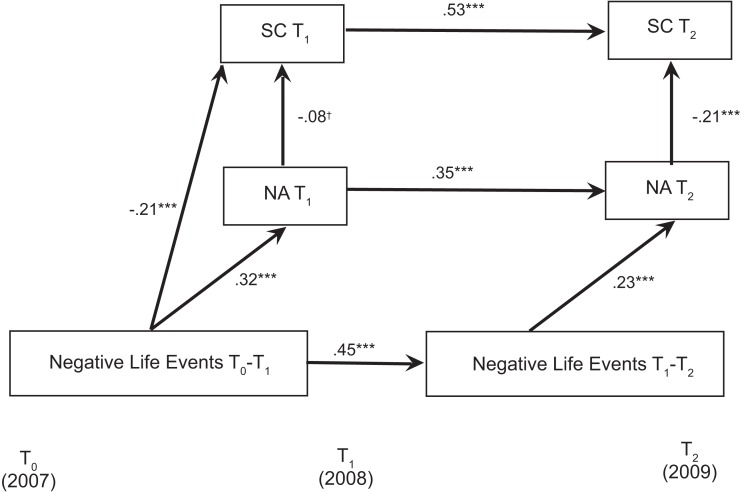
**Path model demonstrating that changes in negative life events are associated with increases in negative affect, which in turn are associated with decreases in self-control in Study 2**. Gender, ethnicity, age, log-transformed income, and school were included as covariates in the model but are not shown. Non-significant paths are also not shown. Life events that occurred during the 1-year period prior to the assessment were reported. NA, negative affect; SC, self-control. ^†^*p* = 0.056. ***p < 0.001.

As predicted, the effect of changes in negative life events on changes in self-control was mediated by changes in negative affect (see Figure 2)^3^. Specifically, increases in stressful life events predicted increases in negative affect (β = 0.23, *p* < 0.001), which in turn predicted decreases in self-control, β = −0.21, *p* < 0.001. Consistent with Study 1, negative life events at Time 1 predicted self-control at Time 2 (β = −0.19, *p* < 0.001), and changes in negative life events predicted changes in self-control, β = −0.10, *p* = 0.016^4^. In sum, using alternative measures of negative life events and self-control than were used in Study 1, Study 2 confirmed our first hypothesis: negative life events predicted rank-order decreases in self-control over a 1-year period. Further, Study 2 confirmed our second hypothesis that longitudinal increases in psychological distress during the same period mediated the relationship between negative life events and self-control impairment.

## Study 3

In Studies 1 and 2, we confirmed that increases in negative life events predict decreases in self-control in children over time, and in Study 2, we demonstrated that negative affect mediated this relationship. Study 2, however, had two major limitations. First, one could argue that negative affect is a good proxy for, but not isomorphic with, psychological distress. Second, unlike in Study 1, participants in Study 2 retrospectively reported recent life events on the same day they provided ratings of self-control (on two occasions separated by 1 year). Thus, we could not use temporal precedence to rule out reverse causality. To address these limitations in Study 3, we directly measured psychological distress using the Perceived Stress Scale (Cohen et al., 1983), and we assessed each construct at separate times: negative life events in the fall, perceived stress in the spring, and self-control in the summer.

### Participants

Participants were fifth through seventh grade students at two public schools in the northeast. About 83% of the 561 students elected to participate. Participants in the final sample (*N* = 464) were not significantly different from excluded participants in terms of gender, ethnicity, or age, *p*s > 0.05. At the first data collection in fall 2010, the mean age of the participants was 12.45 years (SD = 1.17). Ninety-four percent of participants were Black, 4% were Hispanic, and 2% were other ethnicities; 50% were female.

### Procedure and measures

Students and teachers completed consent forms and questionnaires in fall 2010. In spring 2011, students filled out the Perceived Stress Scale. In summer 2011, students and teachers completed questionnaires on self-control targeting students.

#### Negative life events

In fall 2010, children indicated which 14 negative life events, 11 of which were taken from the LEC (Johnson and McCutcheon, 1980), occurred in their lives *during the past year*. This measure of negative life events was identical to that we employed in Study 2.

#### Self-control

In fall 2010 and summer 2011, children completed the Impulsivity Scale for Children (ISC; Tsukayama et al., submitted). Separately, two teachers completed an informant version of the ISC. For consistency with the previous studies, we reverse-scored each item so that higher scores denote higher self-control. Observed internal reliability coefficients ranged from α = 0.77 to 0.91, for self-report, and both teacher ratings. In addition, the teachers completed an informant version of the Brief Self-Control Scale (BSCS, Tangney et al., 2004), a 13-item questionnaire that includes domain-general self-control items endorsed on a five-point Likert scale, where 1 = *not like me at all* and 5 = *very much like* (e.g., “This student is good at resisting temptation”). Observed internal reliability coefficients ranged from α = 0.96 to 0.97 for both time points.

In fall 2010, intercorrelations among self-report and teacher ratings of self-control ranged from *r*s = 0.27 to 0.85, *p*s < 0.001. In summer 2011, intercorrelations among self-report and both teacher ratings of self-control ranged from *r*s = 0.27 to 0.81, *p*s < 0.001. We created separate composite self-control scores for fall 2010 and summer 2011 by averaging the standardized scores of the self and teacher ISC measures, averaging the standardized scores for the teacher BSCS measures, then averaging these ISC and BSCS composites. Following Nunnally (1978), we found the internal reliability of composite self-control measures to be 0.97 for both fall 2010 and summer 2011. On average across the two time points, approximately 86% of participants had all five ratings, 8% had four, 2% had three, and 4% were missing all five of the ratings. We averaged the non-missing scores for participants who were missing one to three scores, and we used the single non-missing score for participants who were missing four scores.

#### Perceived stress scale

In spring 2011, children completed four items from the Perceived Stress Scale (Cohen et al., 1983), which asks how unpredictable, uncontrollable, and overloaded respondents find their lives (e.g., “How often have you felt that you were unable to control the important things in your life” and “How often have you felt difficulties were piling up so high that you could not handle them”) in the last month on a five-point scale ranging from 1 = *never* to 5 = *very often*. The observed internal reliability coefficient was α = 0.53.

#### Socioeconomic status and demographic variables

We obtained data on gender, ethnicity, birthdate, and home addresses from school records. Using home addresses in conjunction with U.S. Census bureau data, we estimated the family income for each participant and used this estimate as a measure of socioeconomic status.

### Results and discussion

Summary statistics and bivariate correlations are provided in Table 3. On average, children reported between 4 and 5 negative life events per year. The 1-year test-retest stability of self-control was *r* = 0.81, *p* < 0.001.

**Table 3:** **Summary statistics and bivariate correlations in Study 3**.

Variables	*M*	*SD*	1	2	3	4	5	6	7	8	9	10
1. Negative life events T_0_–T_1_ (fall 2009–fall 2010)	4.31	2.60	–									
2. Perceived stress T_2_ (spring 2011)^a^	2.65	0.78	0.13**	–								
3. Self-control T_1_ (fall 2010)	−0.01	0.83	−0.09	−0.08	–							
4. Self-control T_3_ (summer 2011)	−0.01	0.82	−0.10*	−0.15**	0.81***	–						
5. Age in fall 2010	12.45	1.17	0.11*	0.04	0.02	0.03	–					
6. Income	$33,450	$14,453	−0.09	−0.06	0.05	0.03	0.03	–				
7. School	44%		−0.09	0.03	−0.09	−0.05	−0.24***	−0.01	–			
8. Female	50%		0.10*	0.14**	0.23***	0.24***	0.02	−0.04	0.04	–		
9. Black	94%		0.07	0.07	−0.14**	−0.14**	−0.03	0.03	0.08	0.05	–	
10. Hispanic	4%		−0.07	−0.07	0.14**	0.15**	0.04	−0.09	−0.11*	−0.05	−0.85***	–
11. Other	2		−0.02	−0.02	0.03	0.01	0.00	0.09	0.03	−0.02	−0.50***	−0.03

^a^Summary statistics are based on raw scores. SEM results use a latent variable for perceived stress. *p < 0.05. **p < 0.01. ***p < 0.001.

**Figure 3:**
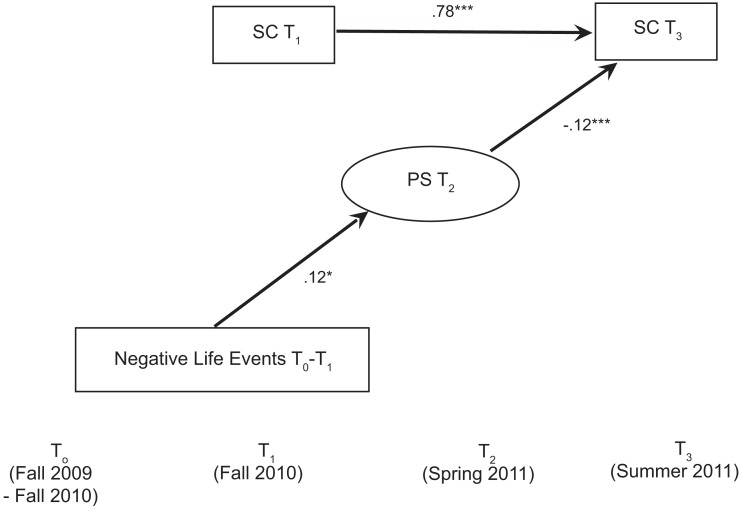
**Structural equation model demonstrating that negative life events predict perceived stress, which in turn predicts decreases in self-control in Study 3**. Gender, ethnicity, age, log-transformed income, and school were included as covariates in the model but are not shown. Non-significant paths are also not shown. Life events that occurred during the 1-year period prior to the assessment were reported. PS, perceived stress; SC = self-control. *p ≤ 0.05. ***p ≤ 0.001.

We fit a structural equation model to test whether perceived stress mediates the effect of negative life events on decreases in self-control controlling for school site, gender, ethnicity, age, and socioeconomic status. About 9% of children were missing data on one or more variables; therefore, we used FIML to estimate our models. Because of the perceived stress scale’s relatively modest internal reliability (α = 0.53), we used a latent variable to adjust for measurement error. Thus, we used a structural equation model instead of path models as in Studies 1 and 2. As shown in Figure 3, the model fit the data well, χ^2^(29) = 40.31, *p* = 0.079; CFI = 0.98; RMSEA = 0.03 (90% confidence interval = 0.00 to 0.05).

As predicted, the effect of negative life events on rank-order decreases in self-control over the school year was mediated by perceived stress. Specifically, negative life events assessed retrospectively in the fall predicted perceived stress in the spring (β = 0.12, *p* = 0.05), which subsequently predicted rank-order decreases in self-control from fall to summer, β = −0.12, *p* = 0.001. In sum, psychological distress, as measured by perceived stress, mediated the relationship between prior reports of negative life events and subsequent decreases in self-control.

## General Discussion

The present investigation is the first to examine the prospective, longitudinal impact of negative life events on ratings of self-controlled behavior during the important developmental stage of early adolescence. In three large samples of young adolescents, recently experienced negative life events predicted lower self-control. These effects were small in magnitude but reliable, holding when controlling for baseline self-control (in all studies), prior reports of negative life events (in Studies 1 and 2), and a rich set of demographic covariates (in all studies). Similarly, these findings held when negative life event checklists were completed by mothers (in Study 1) or children (in Study 3) on a separate occasion, at least 6 months prior to the completion of self-control questionnaires by three different raters (in Studies 1 and 3). As predicted, the effects of objective negative life events on self-control were mediated by subjective reports of psychological distress (in Studies 2 and 3). Collectively, the current findings confirm that the stressors school-age children typically encounter in real life can indeed precipitate measurable impairments in self-control observable to parents, teachers, and the children themselves. Thus, despite substantial rank-order stability in self-control across development (a pattern we replicated in each of our three studies), situational influences can play an important role in determining children’s ability to regulate their attention, behavior, and emotion.

Our investigation relied on non-experimental data. Although this constrains our ability to draw causal inferences, it is notable that the observed relations held when controlling for a range of likely confounds, including socioeconomic status, baseline self-control, and prior negative life events. In addition, observed relations were not likely an artifact of common method variance because multiple raters were used to assess self-control. Furthermore, in Study 2, we examined the effect of *changes* in negative life events on *changes* in psychological distress and, in turn, the effect of these changes on *changes* in self-control. This design minimizes the likelihood that the observed relations among life events, affect, and self-control reflect dispositional characteristics (e.g., a tendency to report or experience more negative life events as well as higher levels of negative affect). Finally, in Study 3, negative life events, perceived stress, and self-control were assessed at separate time points. In sum, numerous features of our study design and statistical analyses strengthen the internal validity of this investigation.

The present findings provide indirect support for dual-process theories of self-control (Metcalfe and Mischel, 1999; Steinberg, 2004; Loewenstein and O’Donoghue, 2007; Carver et al., 2009; Hofmann et al., 2009). In the developmental literature, for example, the *hot/cool model* (Metcalfe and Mischel, 1999; Mischel and Ayduk, 2004) contrasts two opponent systems: the *cool system* is described as affectively-neutral, flexible, slow, and strategic, whereas the *hot system* is characteristically impulsive and reflexive, responding quickly to salient trigger features, either appetitive or affective in nature, of immediately available stimuli. The balance of activity between hot and cool processes has been postulated to be modulated by perceived stress: “as the stress level increases, the cool system becomes increasingly dysfunctional, leaving the hot system to dominate processing” (Metcalfe and Mischel, 1999, p. 8). From an evolutionary perspective, it makes sense that cool, goal-directed processes are dominant when the environment is stable and safe. Contrariwise, impulsive responding may have increased chances of survival in the face of uncertainty and peril:

At low levels of stress, it is to the organism’s advantage to take in as much information as possible and to store it in a neutral manner for later remembrances and uses. This allows for complex thinking, planning, and remembering. However, when the stress level is high–conditions in which an animal may be under threat for its life – quick responding driven by innately determined stimuli or stimuli that have been conditioned to produce immediate responding is essential (Metcalfe and Mischel, 1999, p. 8).

To date, empirical support for these predictions has come from animal studies or human laboratory studies of acute mild stress on performance tasks (see Arnsten, 2009 for a review). The current findings provide direct evidence that children are indeed more impulsive when negotiating stressful life circumstances, a response which may be maladaptive in contemporary society but may have increased the likelihood of survival for most of human history (Arnsten, 1999).

### Limitations and future directions

Some limitations of the current study suggest profitable directions for future research. First, as Monroe (2008) has noted, interview-based methods provide more reliable and valid measures of life events than the self-report checklists we used in the current investigation. It seems likely to us that the observed associations between negative life events and changes in self-control, which were small in size, might have been underestimated as a consequence of our relatively crude predictor measure.

Second, further investigation is needed to test the generalizability of our findings across development. We conducted exploratory moderation analyses (results not shown) examining age, gender, socioeconomic status, baseline self-control, and baseline life events (using Bonferroni corrections) within each study and found none of these interaction effects were significant, *p*s > 0.10. However, the participants in our investigation were similar in developmental stage. Thus, studies including younger children, older adolescents, and adults of various ages are needed to fully explore age as a potential moderator. More generally, future research should explore moderators of the relationship between negative life events and self-control. Important work in this direction has already demonstrated that the impact of negative life events on outcomes other than self-control is moderated by genetic polymorphisms (Caspi et al., 2003), coping mechanisms (Compas et al., 2001), social support (Taylor, 2007), and temperament (Mezulis et al., 2006).

Finally, we hope that future work will investigate potentially reciprocal, dynamic relations among negative life events, psychological distress, and self-control over time. The current investigation shows that the experience of stress can impair self-control. Poor self-control is known to cause a wide range of negative life outcomes (e.g., poor performance in school, drug and alcohol use). In the current investigation, we were primarily interested in testing the association between life events and changes in self-control. We therefore deliberately eliminated from our measures events which could reasonably be construed as direct consequences (or indications) of self-control. However, it seems possible that negative life events might in fact precipitate a vicious cycle whereby stressful life events lead to decreases in self-control, which lead to increases in certain types of stressful life events (e.g., school failure), which further impair self-control, and so on. Such a positive feedback loop has been observed between stressful life events and major depression in children (Kendler et al., 1999) and internalizing and externalizing behaviors in adolescents (Kim et al., 2003).

## Conclusion

The current investigation suggests that stressful life events can impair self-control in adolescents and, further, that this relation is mediated by increased psychological distress. These findings were consistent across three independent, large, and collectively diverse samples of early adolescents in which a range of likely confounds were measured and controlled. Should future research affirm these findings, we see two practical implications. First, educators and clinicians might be informed about the acute influence of negative life events on self-control so as to obviate misinterpretation of stress-related impulsive behavior (e.g., inaccurate diagnoses of attention deficit hyperactivity disorder; Arnsten, 1999). Second, we should identify children who are coping with stressful life events and make available interventions that either directly enhance self-control capacity (e.g., Diamond et al., 2007; Duckworth et al., 2011; Kross et al., 2011) or improve coping skills (e.g., Gillham et al., 2007). Since life stress is a correlate and consequence of social disadvantage, such policies may disproportionately benefit the least advantaged youth in society (Huston et al., 2005; Blair and Raver, 2012).

## Conflict of Interest Statement

The authors declare that the research was conducted in the absence of any commercial or financial relationships that could be construed as a potential conflict of interest.

## Footnotes

^1^We use the term self-control throughout this article but recognize that alternative terms, including self-discipline, self-regulation, and effortful control, are used by other authors to denote the same concept (Duckworth and Kern, 2011).

^2^Controlling for prior levels of a variable enables examination of changes. Because levels at time 1 are statistically held constant, then differences at time 2 are differences in change (Fleeson, 2007). For a more mathematical explanation, see Kessler and Greenberg (1981).

^3^Although there are several ways to test the significance of a mediating effect (e.g., the Sobel test, bootstrapping, etc.), according to MacKinnon et al. (2002), “The best balance of Type I error and statistical power across all cases is the test of the joint significance of the two effects comprising the intervening variable effect” (p. 83).

^4^These estimates were obtained from (1) a model that only included covariates, negative life events at Time 1, and self-control at Time 2, and (2) a model that included covariates, and negative life events and self-control at both time points but did not include negative affect, respectively.
